# Supplementary material for: The feasibility of virtual reality therapy for upper extremity mobilization during and after intensive care unit admission
Source: PeerJ. 2025 Jan 9;13:e18461. doi: 10.7717/peerj.18461 (PMC11725268; doi:10.7717/peerj.18461)
Supplement: Supplemental Information 1 — (A) Absolute hand-grip strength of right hand; (B) Absolute hand-grip strength of left hand; (C) Relative hand-grip strength of left hand. [file peerj-13-18461-s001.pdf]

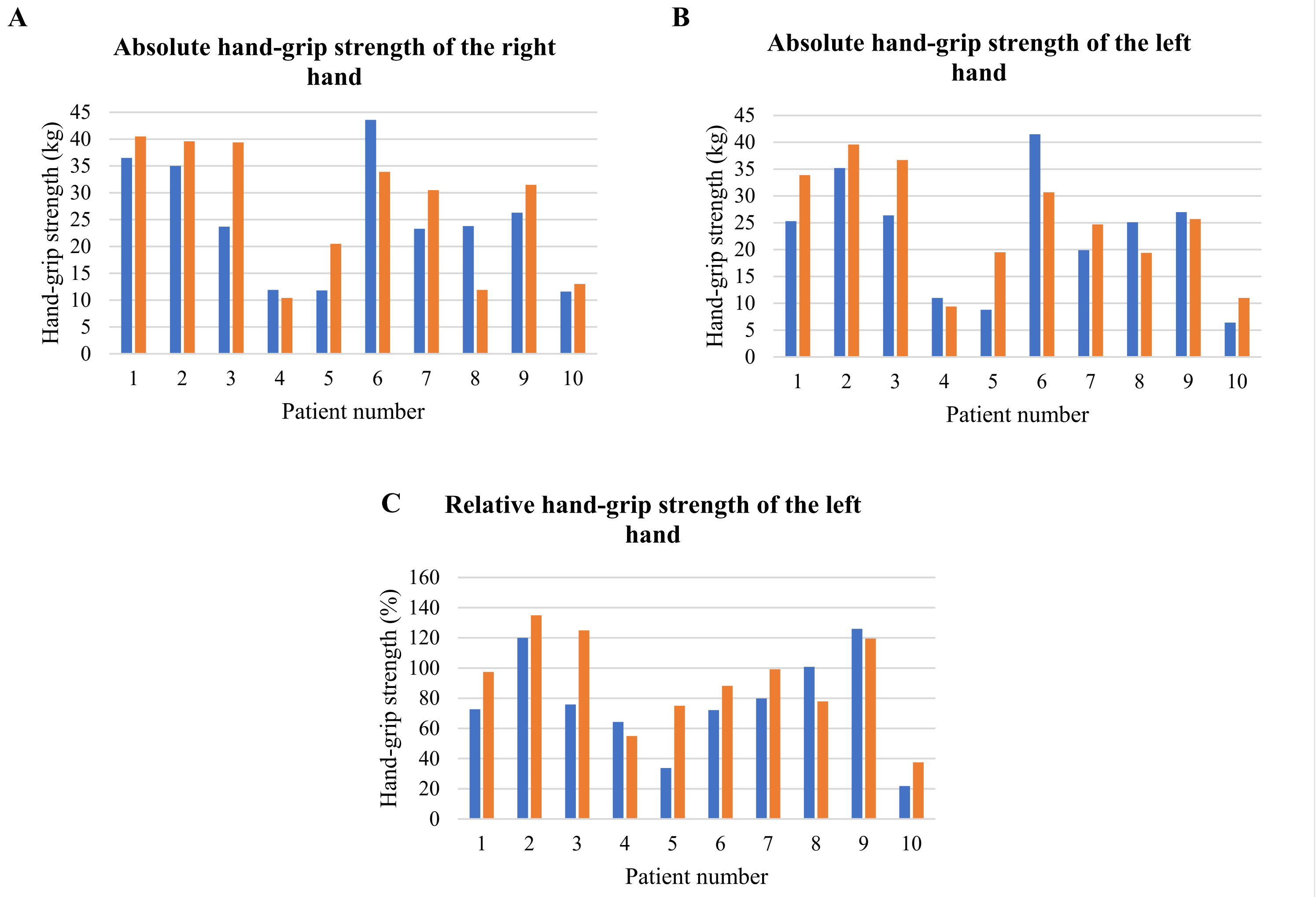
A
Absolute hand-grip strength of the right hand
Hand-grip strength (kg)
0 5 10 15 20 25 30 35 40 45
1 2 3 4 5 6 7 8 9 10
Patient number
B
Absolute hand-grip strength of the left hand
Hand-grip strength (kg)
0 5 10 15 20 25 30 35 40 45
1 2 3 4 5 6 7 8 9 10
Patient number
C
Relative hand-grip strength of the left hand
Hand-grip strength (%)
0 20 40 60 80 100 120 140 160
1 2 3 4 5 6 7 8 9 10
Patient number
